# Modifications of the 7-Hydroxyl Group of the Transthyretin Ligand Luteolin Provide Mechanistic Insights into Its Binding Properties and High Plasma Specificity

**DOI:** 10.1371/journal.pone.0153112

**Published:** 2016-04-06

**Authors:** Lina Nilsson, Andreas Larsson, Afshan Begum, Irina Iakovleva, Marcus Carlsson, Kristoffer Brännström, A. Elisabeth Sauer-Eriksson, Anders Olofsson

**Affiliations:** 1 Department of Medical Biochemistry and Biophysics, Umeå University, SE-901 87, Umeå, Sweden; 2 Department of Chemistry, Umeå University, SE-901 87, Umeå, Sweden; 3 Swedish Defence Research Agency, CBRN Defence and Security, SE-906 21, Umeå, Sweden; Consejo Superior de Investigaciones Cientificas, SPAIN

## Abstract

Amyloid formation of the plasma protein transthyretin (TTR) has been linked to familial amyloid polyneuropathy and senile systemic amyloidosis. Binding of ligands within its natural hormone binding site can stabilize the tetrameric structure and impair amyloid formation. We have recently shown that the flavonoid luteolin stabilizes TTR in human plasma with a very high selectivity. Luteolin, however, is inactivated *in vivo* via glucuronidation for which the preferred site is the hydroxy group at position 7 on its aromatic A-ring. We have evaluated the properties of two luteolin variants in which the 7-hydroxy group has been exchanged for a chlorine (7-Cl-Lut) or a methoxy group (7-MeO-Lut). Using an *in vitro* model, based on human liver microsomes, we verified that these modifications increase the persistence of the drug. Crystal structure determinations show that 7-Cl-Lut binds similarly to luteolin. The larger MeO substituent cannot be accommodated within the same space as the chlorine or hydroxy group and as a result 7-MeO-Lut binds in the opposite direction with the methoxy group in position 7 facing the solvent. Both 7-Cl-Lut and 7-MeO-Lut qualify as high-affinity binders, but in contrast to luteolin, they display a highly non-specific binding to other plasma components. The binding of the two conformations and the key-interactions to TTR are discussed in detail. Taken together, these results show a proof-of-concept that the persistence of luteolin towards enzymatic modification can be increased. We reveal two alternative high-affinity binding modes of luteolin to TTR and that modification in position 7 is restricted only to small substituents if the original orientation of luteolin should be preserved. In addition, the present work provides a general and convenient method to evaluate the efficacy of TTR-stabilizing drugs under conditions similar to an *in vivo* environment.

## Introduction

Transthyretin-related hereditary amyloidosis is linked to more than 100 known heterozygous mutations in the gene coding for transthyretin (TTR) [1], a plasma protein responsible for transporting thyroxine (T4) and holo-retinol-binding protein [2]. The mutated TTR misfolds and misassembles into various intermediate species and amyloid fibrils causing systemic organ dysfunction, frequently including peripheral neuropathy, cardiomyopathy, and ophthalmopathy [3]. TTR-related hereditary amyloidosis is divided into two categories depending on the tissue that is predominantly affected: familial amyloid polyneuropathy (FAP), which mainly affects the peripheral nervous system, and familial amyloid cardiomyopathy (FAC), which predominantly affects the heart [4]. In addition, a late-onset variant of TTR amyloidosis, senile systemic amyloidosis (SSA), is associated with wild-type protein resulting in deposits mainly in the heart [5].

From a mechanistic point of view, the mutations in TTR frequently result in destabilization of the native tetrameric structure of TTR and increase its dissociation into monomers [6]. These monomeric species then misfold and aggregate into amyloid fibrils within various tissues depending on the mutation [7,8].

Since the 1990s, liver transplantation has been used as a treatment for patients with TTR-related hereditary amyloidosis and as a result eliminating the main source of mutated TTR [9]. This treatment is however only suitable for a subset of patients, mainly those with the V30M FAP mutation, and in some cases the disease still progresses post-operatively due to a continuous deposition of the wild-type TTR [10]. In addition, a liver transplant is a major surgery and is associated with many risks due to the life-long immunosuppression treatment that follows transplantation.

The discoveries that dissociation of TTR into monomers is required for amyloid fibril formation [7,8] and that small-molecule ligands increase the activation energy associated with tetramer dissociation [11,12] paved the way for a novel therapeutic approach. Small-molecule ligands that bind within the T4 binding sites of TTR can efficiently stabilize the tetramer [13], and the effect on T4 metabolism is minor because the primary carrier of T4 in plasma is thyroxine-binding globulin and only a very small portion of TTR (<1%) binds to T4 [14]. In 2011, the drug tafamidis, a compound with high ability to bind and stabilize the tetrameric fold of TTR, was approved by the European Medical Agency for treatment of TTR-related hereditary amyloidosis patients with stage I polyneuropathy [15]. However, a beneficial effect could not be confirmed in patients with a more advanced stage of disease [16]. In 2013, diflunisal, a non-steroid anti-inflammatory drug (NSAID) was evaluated, and it was shown that the drug managed to reduce the rate of progression of neurological impairment and preserve the quality of life compared with placebo [17]. Due to the potential side effects, e.g. gastrointestinal bleeding, renal dysfunction, and hypertension frequently observed from long-term use of NSAIDs [18–20], there is still a need for more effective drugs without adverse side effects. Furthermore, there is at present no drug approved for treatment of FAC or SSA, and the therapy for most of these patients is constrained to symptomatic relief.

**Fig 1:**
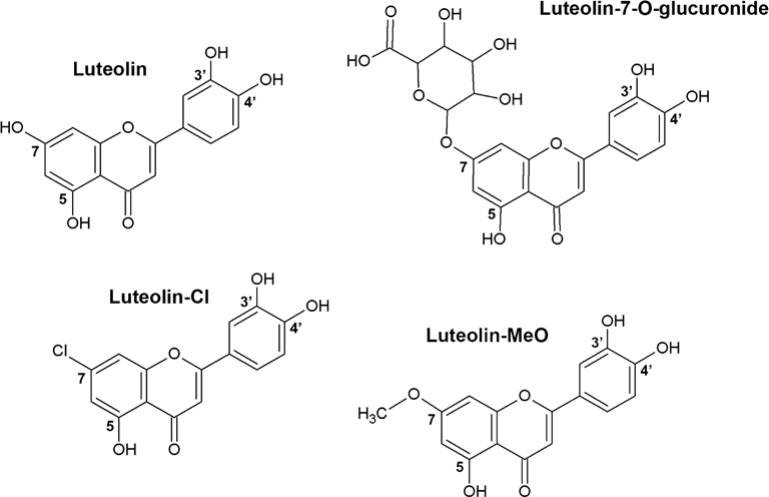
Chemical structures showing luteolin, luteolin-7-O-glucuronide, and the modified variants of luteolin where the 7-OH has been substituted with a chlorine (7-Cl-Lut) or a methoxy group (7-MeO-Lut).

We have previously shown that the flavonoid luteolin is a highly selective binder to TTR in human plasma and that it can attenuate a TTR-mediated cytotoxic effect in cultured neuronal nerve cells and can rescue the phenotype of a *Drosophila melanogaster* FAP model [21]. Luteolin, commonly found in plants and fruits, has a low incidence of side effects [22]. However, in analogy to many drugs luteolin is biotransformed *in vivo*, mainly by phase II enzymes, with glucuronidation and sulfation as the main metabolic pathways [23]. UDP-glucuronosyltransferases (UGTs) are the enzymes responsible for the glucuronidation reaction in which a glucuronic acid moiety is conjugated to the hydroxyls of luteolin. The 7-hydroxy group position is the preferred site for glucuronidation by the human liver (Fig 1) [24].

Our previous studies have shown that a bulky modification in position 7 of luteolin renders an inactive molecule regarding the ability to stabilize TTR [21]. A structural study further confirmed that the 7-hydroxy group is buried at the deep end of the substrate-binding cavity at a site incompatible with incorporation of substantially larger groups [21]. Our aim is to introduce a substituent inert to modification by the UGTs. This limits the options of substituents since frequently used moieties such as carboxyls, thiols and amines all are subjected to glucuronidation [25–27]. In the present work, we have synthesized and analysed *in vitro*, including crystal determinations, two analogues where the hydroxy group at position 7 has been exchanged for a chloro group (7-Cl-Lut) and a methoxy group (7-MeO-Lut), both substituent inert to glucuronidation (Fig 1).

Using an *in vitro* model based on human liver microsomes, we show here how the modifications increase the persistence of the drugs. The crystal structures of TTR complexed to the two analogues show that 7-Cl-Lut binds TTR in a similar manner as luteolin. The larger MeO substituent cannot be accommodated in the same space as the chlorine and as a result 7-MeO-Lut binds in the opposite direction. 7-Cl-Lut and 7-MeO-Lut bind to TTR with a high affinity, however, compared to luteolin both analogues have lost a significant proportion of the high selectivity for TTR in human plasma and display a significantly higher non-specific binding to other components of the plasma.

In this work we also demonstrate how stability measurements in the presence of human plasma, in combination with a liver microsome assay, provide a simple method to evaluate the putative efficacy of TTR-stabilizing drugs *in vivo*.

## Material and Methods

### Synthesis of luteolin analogues

All reactions were carried out under an inert atmosphere with dry solvents, unless otherwise stated. Thin layer chromatography (TLC) was performed on Silica Gel 60 F_254_ using detection with UV light and staining with potassium permanganate or cerium molybdate. Silica gel chromatography was performed with normal phase silica gel (60 Å, 230–400 mesh, Merck, grade 9385). Microwave heated reactions were carried out in sealed Emrys^TM^ process vials with an Initiator^TM^ microwave instrument (Biotage). Temperatures were monitored with an IR probe. The ^1^H and ^13^C NMR spectra were recorded at 298 K with a DRX-400 spectrometer (Bruker) and calibrated using the residual peak of solvent as the internal standard [DMSO-*d*_*6*_ (DMSO-*d*_*5*_ δ_H_ 2.50 ppm, DMSO-*d*_*6*_ δ_C_ 39.5 ppm)]. Low-resolution mass spectrometry was conducted on a Micromass ZQ mass spectrometer with ES^+^ ionization. Preparative HPLC was performed using a VP 250/21 Nucleodur C-18, HTEC, 5 μm column (Macherey-Nagel) on a 333/334 Prep-Scale system with a flow rate of 20 mL/min, detection at 230 nm (Gilson 151), and an CH_3_CN (0.005% HCO_2_H)/H_2_O (0.005% HCO_2_H) eluent system.

#### 2-(2,2-diphenyl-1,3-benzodioxol-5-yl)-5,7-dihydroxy-4H-1-benzopyran-4-one (2)

**Fig 2:**
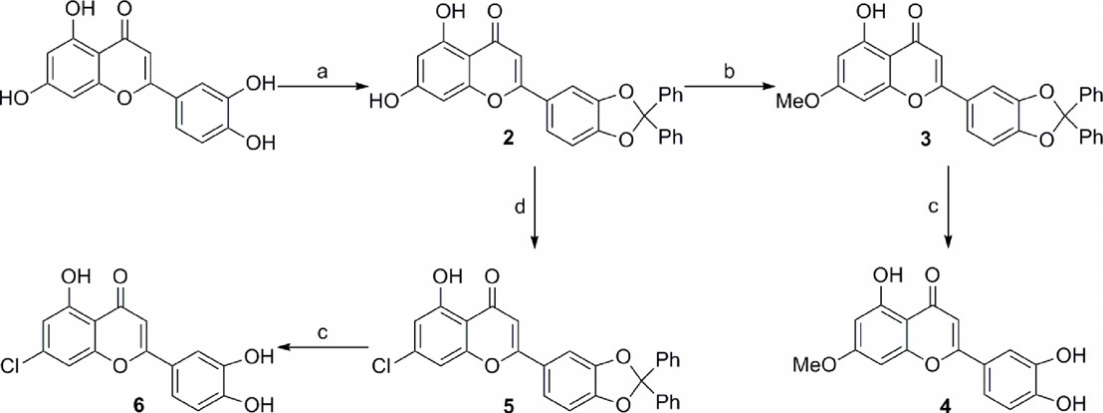
Reagents and conditions: (a) 2.1 equivalents Ph_2_CCl_2_, 2 equivalents DIPEA, Dioxane/NMP, 2 x 30 min, microwave 180°C. (b) 1.2 equivalents MeI, 1.4 equivalents K_2_CO_3_, DMF, 18 h, RT. (c) AcOH/H_2_O 4:1, 1 h, microwave 190°C. (d) 3.3 equivalents POCl_3_, DMF, 5 h, 130°C.

α,α-Dichlorodiphenylmethane (870 mg, 705 μL, 3.67 mmol) and N,N-diisopropylethylamine (DIPEA) (451 mg, 608 μL, 3.49 mmol) were added to a solution of luteolin (1.00 g, 3.49 mmol) and dioxane/NMP 3:1 (20 mL) in a capped vial, and the reaction mixture was heated in a microwave oven for 30 min at 180°C (Fig 2). A second dose of α,α-dichlorodiphenylmethane (870 mg, 705 μL, 3.67 mmol) and DIPEA (451 mg, 608 μL, 3.49 mmol) was added and the reaction was heated for an additional 30 min at 180°C. The resulting mixture was concentrated, and the residue was taken up in CH_2_Cl_2_, washed with NH_4_Cl (sat), NaHCO_3_ (sat) and brine, dried over Na_2_SO_4,_ and concentrated. Purification by column flash chromatography (EtOAc: heptane 1:4 to 1:1) gave **2** as a pale yellow powder (1.30 g, 2.78 mmol, 83% yield).

^1^H NMR (500 MHz, DMSO-*d*_*6*_) *δ* 12.86 (s, 1H), 10.87 (s, 1H), 7.78 (d, *J* = 1.7 Hz, 1H) 7.70 (dd, *J* = 8.3, 1.7 Hz, 1H), 7.57–7.53 (m, 4H), 7.49–7.43 (m, 6H), 7.23 (d, *J* = 8.3 Hz, 1H), 6.89 (s, 1H), 6.51 (d, *J* = 2.1 Hz, 1H), 6.19 (d, *J* = 2.1 Hz, 1H); ^13^C NMR (125 MHz, DMSO-*d*_*6*_) *δ* 181.8, 164.3, 162.8, 161.4, 157.3, 149.5, 147.3, 139.1 (2C), 129.6 (2C), 128.7 (4C), 125.8 (4C), 125.0, 122.2, 117.5, 109.2, 106.9, 104.2, 103.8, 98.9, 94.1. LRMS (ESI) m/z: 451.1 (M+1).

#### 2-(2,2-diphenyl-1,3-benzodioxol-5-yl)-5-hydroxy-7-methoxy-4H-1-benzopyran-4-one (3)

To a stirred solution of **2** (100 mg, 0.22 mmol) in 25 mL dry dimethylformamide (DMF), K_2_CO_3_ (42 mg, 0.30 mmol) was added followed by methyl iodide (37 mg, 16 μL, 0.26 mmol). The resulting solution was allowed to stir for 18 h at 20°C. The reaction mixture was diluted with 100 mL EtOAc and washed with 3 x 100 mL H_2_O and 1 x 100 mL brine. The organic phase was dried (Na_2_SO_4_) and concentrated to give **3** as a crude product which was used in the next step without further purification.

#### 2-(3,4-dihydroxyphenyl)-5-hydroxy-7-methoxy-4H-1-benzopyran-4-one (4)

To the crude product **3** from previous step was added a mixture of acetic acid/water (4:1, 10 mL) in a capped vial. The reaction mixture was heated in a microwave at 190°C for 1 h. The resulting solution was portioned between EtOAc (25 mL) and H_2_O (25 mL), the organic phase washed with NaHCO_3_ sat. (3 x 10 mL), dried with Na_2_SO_4_ and concentrated. The crude product was purified with HPLC using a gradient from 30–100% CH_3_CN/H_2_O (0.005% HCO_2_H) over 40 min, to give **4** as a yellow powder (36 mg, 0.12 mmol, 55% yield over two steps).

^1^H NMR (500 MHz, DMSO-*d*_*6*_) *δ* 12.98 (s, 1H), 9.98 (s, 1H), 9.38 (s, 1H), 7.45 (dd, *J* = 8.3, 2.3 Hz, 1H), 7.44 (d, *J* = 2.3 Hz, 1H), 6.89 (d, *J* = 8.3 Hz, 1H), 6.73 (s, 1H), 6.72 (d, *J* = 2.3 Hz, 1H), 6.37 (d, *J* = 2.3 Hz, 1H), 3.86 (s, 3H); ^13^C NMR (125 MHz, DMSO-*d*_*6*_) *δ* 181.8, 165.1, 164.2, 161.2, 157.2, 149.8, 145.8, 121.4, 119.1, 116.0, 113.5, 104.7, 103.1, 98.0, 92.6, 56.1. LRMS (ESI) m/z: 301.1 (M+1).

#### 2-(2,2-diphenyl-1,3-benzodioxol-5-yl)-5-hydroxy-7-chloro-4H-1-benzopyran-4-one (5)

POCl_3_ (51 mg, 31 μL, 0.33 mmol) was added dropwise to a cooled solution of **2** (45 mg, 0.1 mmol) in DMF (0.5 mL). The reaction mixture was heated to 130°C for 5 h using a microwave before being diluted with EtOAc (10 mL) and washed with H_2_O (3 x 10 mL) and brine (10 mL), dried with Na_2_SO_4_ and concentrated. Filtering through a plug of silica using 1:1 EtOAc:heptane gave a crude product that was used in the next step without further purification.

#### 2-(3,4-dihydroxyphenyl)-5-hydroxy-7-chloro-4H-1-benzopyran-4-one (6)

A mixture of acetic acid/water (4:1, 5 mL) in a capped vial was added to the crude product **5** from the previous step. The reaction mixture was heated in a microwave at 190°C for 1 h. The resulting solution was portioned between EtOAc (15 mL) and H_2_O (15 mL), and the organic phase washed with NaHCO_3_ (sat., 3 x 5 mL), dried with Na_2_SO_4,_ and concentrated. The crude product was purified with HPLC using a gradient from 30–100% CH_3_CN/H_2_O (0.005% HCO_2_H) over 40 min, to give **6** as a yellow powder (15 mg, 0.05 mmol, 50% yield over two steps).

^1^H NMR (500 MHz, DMSO-*d*_*6*_) *δ* 13.09 (s, 1H), 10.10 (s, 1H), 9.42 (s, 1H), 7.48 (dd, *J* = 8.4, 2.3 Hz, 1H), 7.44 (d, *J* = 2.3 Hz, 1H), 7.33 (d, *J* = 1.8 Hz, 1H), 6.91 (d, *J* = 1.8 Hz, 1H), 6.90 (d, *J* = 8.4 Hz, 1H), 6.87 (s, 1H); ^13^C NMR (125 MHz, DMSO-*d*_*6*_) *δ* 182.3, 170.4, 165.0, 160.7, 156.0, 150.3, 145.8, 121.0, 119.5, 116.0, 113.8, 111.2, 108.9, 107.9, 103.6. LRMS (ESI) m/z: 305.0 (M+1).

### Protein expression and purification

Expression of TTR was performed as described previously [28]. Briefly, after transformation of the plasmid into *Escherichia coli* BL21, the cells were grown until an OD600 of 0.6, followed by induction with 0.4 mM isopropyl thiogalactopyranoside at 37°C. Cells were harvested after 18 h and lysed by sonication. The cell debris was removed by centrifugation at 20,000 × g for 30 min, and the supernatant was collected and loaded on an anion exchange column (Q-sepharose, Amersham Biosciences) and eluted with a NaCl gradient. Fractions containing TTR were combined and concentrated using Centriprep filter units with Ultracel YM-10 membranes (Millipore). The concentrate was loaded on a gel filtration column (Superdex G75-16/60, Amersham Biosciences) equilibrated with PBS.

### Selectivity of luteolin, 7-Cl-Lut and 7-MeO-Lut in plasma

We have recently developed an assay in which the relative stability as a function of drug concentration can be evaluated in complex solutions [21]. TTR is found within the human plasma, and it is thus of interest to analyze drug efficacy in TTR’s natural environment.

The analysis was performed as described previously with some modifications [29]. Human plasma from a healthy donor was centrifuged at 20,000 × *g* for 10 min. The plasma was filtered using a 0.45 μm syringe filter (Filtropur S, Nümbrecht, Germany). A solution of 1 M sodium phosphate buffer at pH 7.0 was added to a final concentration of 40 mM. Luteolin, 7-Cl-Lut and 7-MeO-Lut were dissolved in dimethyl sulfoxide (DMSO) and added to the plasma at the indicated concentrations with a final DMSO concentration of 5%. The reactions were incubated for 2 h at 20°C. Denaturation was initiated by addition of 4 M urea, 40 mM sodium phosphate buffer pH 7.4, and 150 mM NaCl. The samples were incubated for 18 h at 20°C followed by separation by gel electrophoresis using tricine-based SDS-PAGE with 0.025% SDS in the running buffer and 0.2% SDS in the loading buffer. SDS at this concentration does not denature TTR tetramers but does prevent re-association of monomers. Gel electrophoresis was followed by western blotting using rabbit anti-TTR antibody (anti-prealbumin, DAKO, Denmark) followed by a horseradish peroxidase-labeled antibody (anti-rabbit HRP, GE Healthcare, Sweden). Blocking of nonspecific binding on the membrane and incubation of the antibodies were performed in 5% fat-free powdered milk containing 0.3% Tween-20. All washing steps were performed in 40 mM Tris, 150 mM NaCl, and 0.3% Tween-20. Bound antibodies were visualized on photographic film using enhanced chemiluminescence (ECL-prime, GE Healthcare, UK). The employed rabbit anti-transthyretin antibody, however, does not detect the native fraction of TTR very well, and as a consequence only the change in monomers, which reflect the level of dissociation, was monitored [21]. The presence of monomers on the photographic film was quantified through a densitometric analysis using the ImageJ 1.48 software (National Institutes of Health, Boston, US), which also ensured that all analyses were performed within a linear range.

### Probing the level of glucuronidation using human liver microsomes

Pooled female human liver microsomes (n = 10) were purchased from XenoTech (Lenexa, US) and stored at −80°C and rapidly thawed before use. The microsomes, 0.1 mg/mL, were activated by incubation with alamethicin (Santa Cruz Biotechnology, US), 50 μg/mL microsomal protein, in 0.1 M sodium phosphate buffer using a VWR Mini Shaker (Radnor, US) at 37°C and 550 rpm for 10 min. Luteolin, 7-Cl-Lut or 7-MeO-Lut was added to give a final concentration of 10 μM and 5% DMSO, and these were pre-incubated with the activated microsomes for 5 min at 37°C and shaking at 550 rpm. The reaction was initiated by addition of 5 mM uridine 5’-diphosphoglucuronic acid, (Santa Cruz Biotechnology, US). The incubation time ranged from 0 min to 5 h. The reactions were quenched by flash freezing and stored at −80°C. The microsomes were heat inactivated using a VWR UnoCycler (Radnor, US) for 5 min at 95°C. All experiments were repeated three times.

In order to calculate depletion kinetics, the percentage of the remaining drug was calculated by comparison with the initial quantity at 0 min. The half-life (t_1/2_) of luteolin, 7-Cl-Lut and 7-MeO-Lut was calculated using GraphPad Prism (GraphPad Software, Inc., US, version 5.0). Data were fitted to one-phase exponential decay:

C=C0*exp(-K*t) (1)

$$\text{The half-life}\;\left({\text{t}}_{1/2}\right)=0.693/\text{K}$$ (2)

### Ultra-high performance liquid chromatography (UHPLC)

Microsome-treated samples were diluted in 50% DMSO. Analytical HPLC was performed using a Nexera UHPLC system (Shimadzu, US) connected to a diode array detector (SPP M20A). Samples were analyzed using a Nucleodur C18 HTec column (EC 150 × 4.6, 5 μm, Macherey-Nagel) with a flow rate of 1 mL/min. Aliquots of 20 μL of each sample were injected, and detection was performed at 345 nm. The mobile phase was composed of solvent A (H_2_O in 0.1% TFA) and solvent B (acetonitrile in 0.1% TFA). The binary gradient profile was as follows with solvent B as the reference: 0–2.5 min, 17% B; 2.5–7.5 min, 17–25% B; 7.5–15.5 min, 25–35% B; 15.5–20.5 min, 35–50% B; 20.5–25.5 min, 50–100% B; 25.5–30.5 min, 100% B; 30.5–31 min, 100–17% B; 31–35.5 min, 17% B. Before first injection, the column was equilibrated at 17% B for 5 min.

### Microscale Thermophoresis (MST)

We used MST to measure the affinity between the different ligands and TTR. TTR was labelled with the Monolith NT protein labelling kit (Nanotemper Technologies, Germany) using the sulfhydryl-reactive red fluorescent dye NT-647 Maleimide according to the manufacturer’s instructions. Labelled TTR was kept at a constant concentration of 300 nM, and luteolin, 7-Cl-Lut and 7-MeO-Lut were serially diluted yielding 13, 16 and 12 respectively different concentrations ranging from 0.24 nM to 1 μM for luteolin, 0.24 nM to 8 μM for 7-Cl-Lut, and 0.61 nM to 1.25 μM for 7-MeO-Lut. Experiments were carried out in phosphate-buffered saline and 5% DMSO with a Monolith NT.115 instrument using standard-treated capillaries (Nanotemper Technologies, Germany). Fluorescence was detected for 5 s, and the laser was switched on for 30 s followed by fluorescence detection for another 5 s. The delay time between runs was set to 25 s. The LED power and the MST were set to 80% for all drugs. Kd values were determined using NTanalysis software (NanoTemper Technologies, Germany) by fitting the data representing the mean ± S.E.M. from three independent measurements to the Kd fit model.

### Crystallization of TTR-7-Cl-Lut and TTR-7-MeO complexes

The TTR protein was crystallized as described previously [21]. Briefly, purified TTR was dialyzed against 10 mM sodium phosphate buffer pH 7.6 and 100 mM KCl and concentrated to 5 mg/mL using an Amicon Ultra centrifugal filter device (Millipore, 3 kDa molecular-weight cutoff) and co-crystallized at room temperature with a 5 molar excess of 7-Cl-Lut or 7-MeO-Lut using the vapor-diffusion hanging drop method. A drop containing 3 μL protein solution was mixed with 3 μL of the reservoir solution and equilibrated against 1 mL of reservoir solution containing a range of 1.3–1.6 M sodium citrate and 3.5% *v/v* glycerol at pH 5.5 in 24-well Linbro plates. Crystals grew to dimensions of 0.2 × 0.1 × 0.4 mm^3^ after 5 days. The crystals were cryoprotected with 12% *v/v* glycerol.

### Data collection, integration, and structure determination

The X-ray diffraction data of the TTR-7-Cl-Lut and TTR-7-MeO-Lut complexes were collected under cryogenic conditions to 1.25 Å and 1.35 Å resolution respectively at the European Synchrotron Radiation Facility in Grenoble, France, on beamline ID29 using Pilatus 6M detectors. The diffraction data were processed with XDS [30] and scaled using AIMLESS from the CCP4 software suite [31]. The X-ray model of TTR (PDB ID: 1F41) [32] and X-ray data from 40.0–2.5 Å resolution were used in molecular replacement searches with the program PHASER [33]. The 7-Cl-Lut and 7-MeO-Lut molecules are best defined in the B-B´ hormone binding site cavity. The models were refined against all the diffraction data using PHENIX REFINE [34]. Manual map inspections were performed with COOT [35]. During refinement, isotropic B-factors were first applied followed by anisotropic B-factors at the end of the refinement using all data. Molecular graphics were produced using CCP4mg [36]. See Table 1 for details of the data collection and refinement statistics. Structure factors and coordinates of the TTR–7-Cl-Lut and TTR–7-MeO-Lut complexes have been deposited at the protein data bank (PDB ID: 5EN3 and 5IHH, respectively).

**Table 1:** Data collection and refinement statistics for the TTR-7-Cl-Lut and TTR-7-MeO-Lut-complexes.

Data-collection statistics	TTR-7-Cl-Lut	TTR-7-Cl-Lut (Anomal.)	TTR-7-MeO-Lut
Wavelength (Å)	0.914	1.542	0.976
Space group	P21212	P21212	P21212
Unit-cell parameters (Å)	a = 43.0, b = 85.5, c = 64.2	a = 42.5, b = 85.8, c = 63.7	a = 42.9, b = 85.9, c = 63.8
Resolution limits (Å)	40.0–1.25 (1.29–1.25)	40.0–1.60 (1.65–1.60)	43.0–1.35(1.37–1.35)
Total No. of reflections	597380	258173	376548
No. of unique reflections	71090 (6911)	31330 (3023)	52635(2670)
Multiplicity	9.0 (8.9)	10.1 (6.8)	7.2(7.3)
Completeness (%)	99.5 (97.8)	99.8 (98.1)	100.0(100.0)
R_sym_	0.044 (0.541)	0.049 (0.340)	0.047(0.617)
<I/σ (I)>	21.66 (3.21)	18.34 (3.38)	19.1(3.1)
CC1/2	0.999 (0.907)	1.000 (0.989)	0.999(0.852)
**Refinement and model building statistics**			
Resolution Range (Å)	40.00–1.22		43.0–1.35
R factor (%)	13.6 (18.5)		14.9(16.7)
R free (%)	15.8 (22.5)		16.7(21.4)
No. of protein atoms	2133		1961
No. of water molecules	221		257
No. of ligand atoms /glycerol atoms /sodium ion	42/6/1		44/0/1
**Rms deviations from ideal geometry**			
Bond length (Å)	0.011		0.009
Bond angles (°)	1.36		0.99
**Ramachandran plot**			
Residues in most favored regions (%)	99.4		98.0
Residues allowed regions (%)	0.8		2.0
Residues in disallowed regions (%)	0.0		0.0
Average B-factor (Å^2^)	23.7		21.7
PDB code	5EN3		5IHH

## Results

### Determination of binding affinities between the drugs and TTR

**Fig 3:**
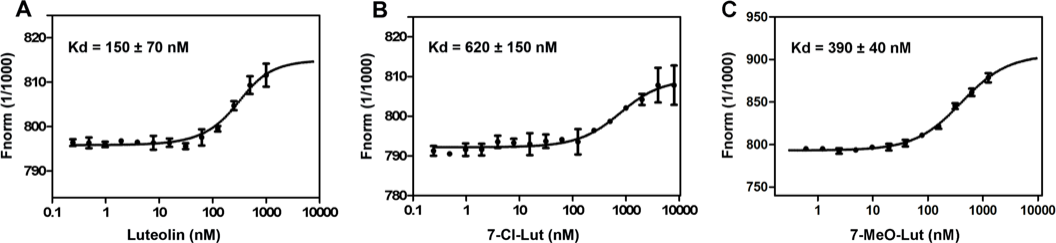
Binding of luteolin, 7-Cl-Lut and 7-MeO-Lut to NT-647–labeled TTR. Various concentrations of the compounds were titrated against 300 nM of NT-647–labeled TTR. Kd values were determined by averaging three independent experiments (mean ± S.E.M.) using the NT analysis software. The thermophoresis signal was plotted against luteolin (A), 7-Cl-Lut (B) and 7-MeO-Lut (C) concentrations. The Kd values were determined to be 150 ± 70 nM for luteolin, 620 ± 150 nM for 7-Cl-Lut and 390 ± 40 nM for 7-MeO-Lut.

In order to obtain binding affinities between the drugs and TTR, we employed microscale thermophoresis (MST), which is a technique based on the directed movement of molecules in a temperature gradient. This phenomenon depends on a variety of molecular properties such as size, charge, hydration shell, and conformation. The dissociation constant (Kd) for luteolin was determined to be 150 ± 70 nM, which is in accordance with the previously published value of 100 nM determined by isothermal titration calorimetry [37] (Fig 3A). The Kd values for 7-Cl-Lut and 7-MeO-Lut were determined to be 620 ± 150 nM and 390 ± 40 nM respectively (Fig 3B and 3C).

### Crystal structures of the TTR-7-Cl-Lut and TTR-7-MeO-Lut complexes

**Fig 4:**
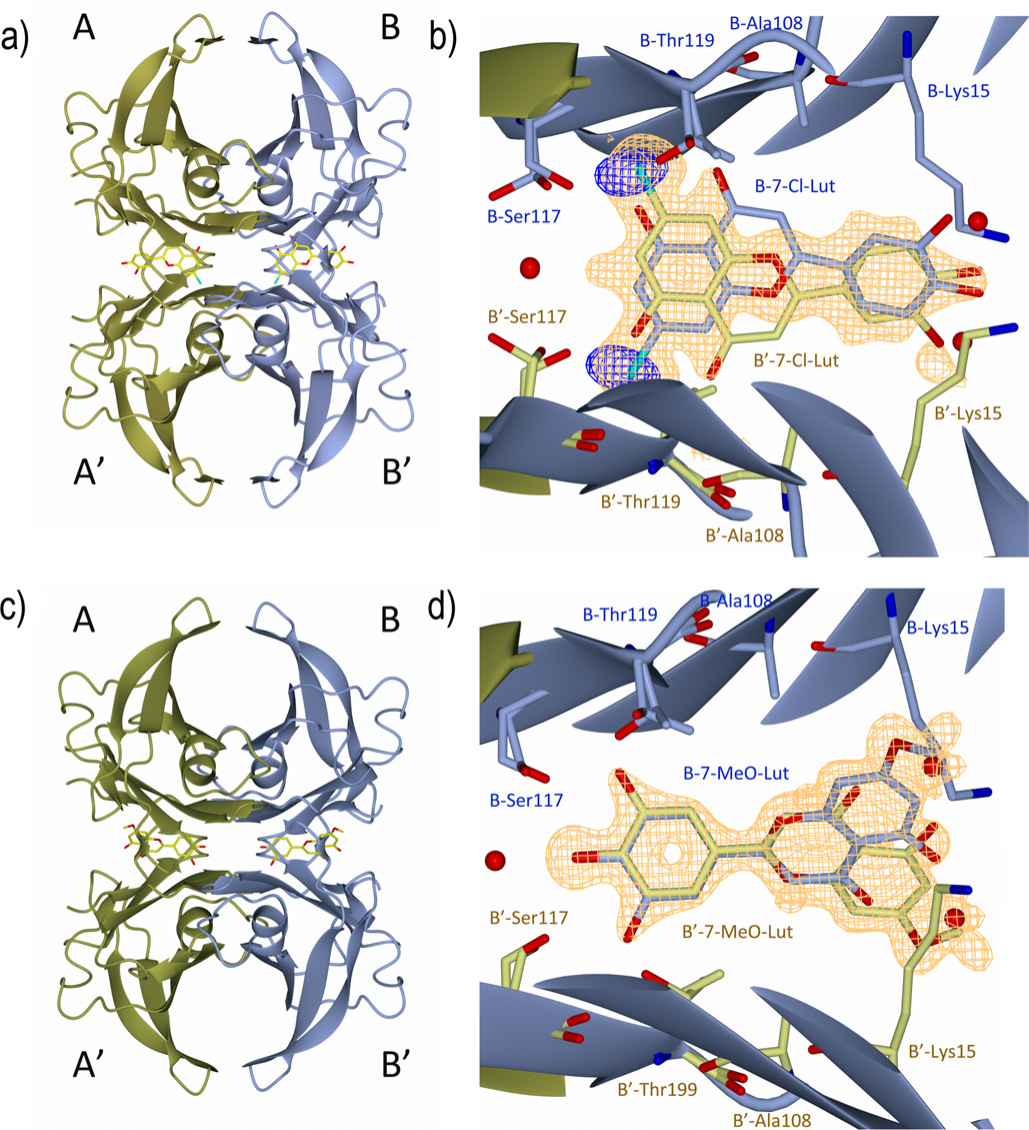
A and C) The TTR monomers in the tetrameric structure are shown as ribbons and are labeled A (gold) and B (ice-blue). The symmetry-related monomers are labeled A´ and B´, respectively. Two 7-Cl-Lut molecules (shown in A) and two 7-MeO-Lut molecules (shown in C) bind at the T4 hormone binding pocket and are shown as sticks. (B) The quality of the electron density map at the BB´ dimer-dimer interface of 7-Cl-Lut. The σA-weighted (m|Fo|-D|Fc|) electron density map contoured at 3 times the root-mean-square value of the map is shown in orange. The anomalous log-likelihood-gradient (LLG) map shown in dark blue shows unambiguously the positions of the two symmetry-related chlorine atoms and verifies the modeled orientation of the 7-Cl-Lut compound in the binding site. (D) The quality of the difference Fourier electron density map at the BB´ dimer-dimer interface of 7-MeO-Lut, calculated as described in (B). To reduce model bias, the 7-Cl-Lut and 7-MeO-Lut molecules were excluded from the coordinate files that were subjected to one round of simulated annealing before calculation of the electron density map.

We have recently solved the structure of the TTR–luteolin complex, which shows how the aromatic A-ring of luteolin is inserted into the deep end of the hydrophobic T4-hormone binding site [21]. Structural determination of the TTR-7-Cl-Lut complex reveals that 7-Cl-Lut binds in the same position as luteolin. The TTR-7-MeO-Lut complex however shows that the 7-MeO-Lut ligand is oppositely oriented in the binding site (Fig 4). The binding of 7-Cl-Lut at the deep end of the cavity was verified with anomalous difference maps (Fig 4B), and the reversed binding of 7-MeO-Lut is evident from difference maps (Fig 4D). Similar to the TTR-luteolin complex structure, no direct hydrogen bonds are formed between the Nz atom of Lys15 and the luteolin analogues. The methoxy group is however wedged in the hydrophobic pocket formed by Lys15, Thr106 and Val121.

**Fig 5:**
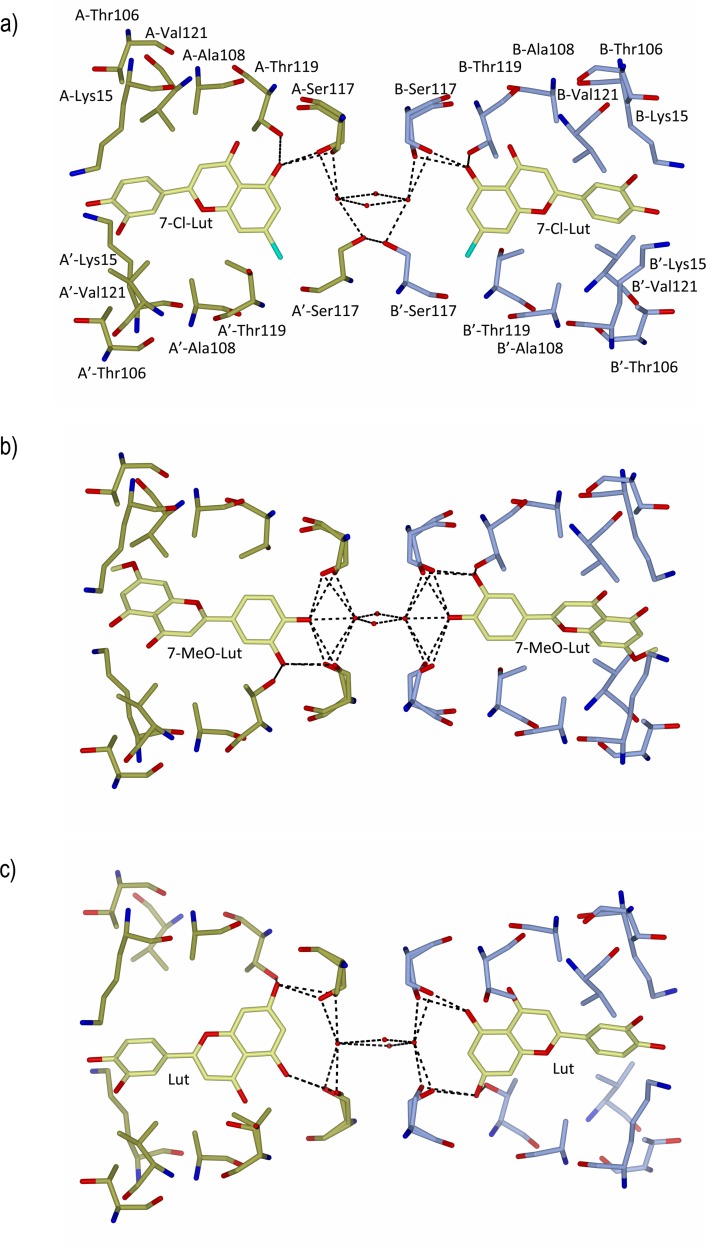
Comparison between TTR-binding sites of A) 7-Cl-Lut, B) 7-MeO-Lut and C) luteolin in the dimer-dimer interface. The figures show binding of one compound in the AA’ and BB’ binding sites. The binding of 7-Cl-Lut at the AA’ and BB’ channel is coupled, i.e. if a 7-Cl-Lut ligand binds at the AA’ site with the chlorine directed down as shown in A, the 7-Cl-Lut ligand binding at the BB’ site must have an orientation so that its chlorine atom is also pointing down. For 7-MeO-Lut (B) and luteolin (C) the orientation of the ligands is not coupled. Hydrogen bonds are indicated with dotted lines. Carbon atoms from TTR and ligands are colored with blue and yellow bonds, respectively. Residue labels are only shown in A).

Exchange of the hydroxy group at position 7 of luteolin for a chloro or methoxy group has structural consequences in both cases, and for example, impairs the luteolin analogues ability to form bridging hydrogen bonds to symmetry-related Ser117 and Thr119 over the dimer-dimer interface (**Fig 5**).

### Substitution of the 7-OH group improves metabolic stability

**Fig 6:**
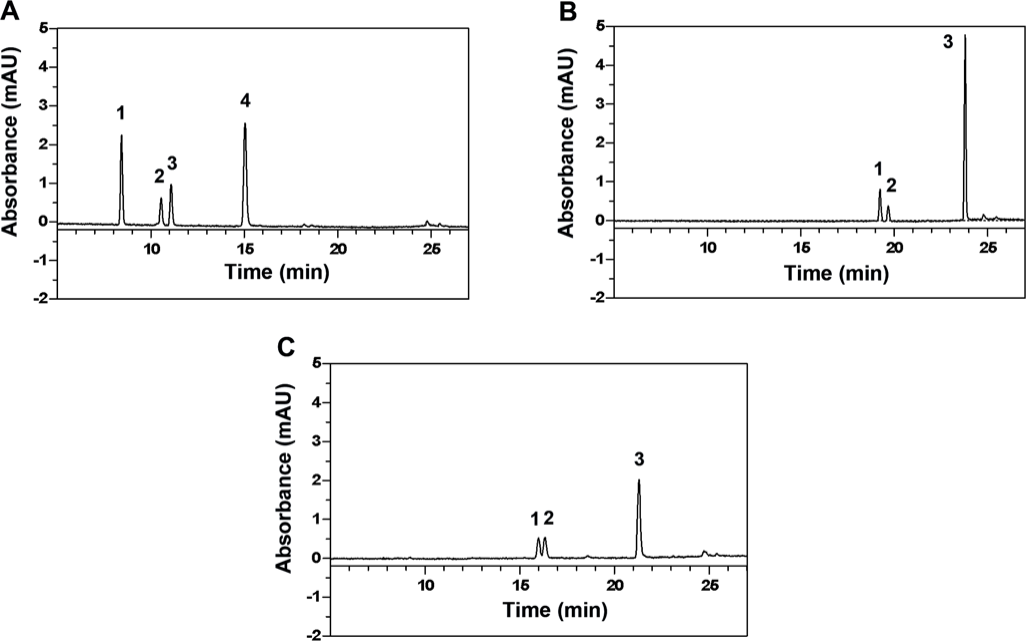
UHPLC chromatogram of luteolin compounds incubated with 0.1 mg/mL human liver microsomes after 15 min incubation at 37°C. Detection was at 345 nm. A) Luteolin and its metabolites. 1: luteolin-7-O-glucuronosyl, 2: luteolin-4’-O-glucuronosyl, 3: luteolin-3’-O-glucuronosyl [24], 4: luteolin. B) 7-Cl-Lut and its metabolites. 1: luteolin-Cl-4’-O- glucuronosyl and 2: luteolin-Cl-3’-O-glucuronosyl. 7-Cl-Lut is peak 3. C) 7-MeO-Lut and its metabolites. 1: luteolin-MeO-4’-O-glucuronosyl and 2: luteolin-MeO-3’-O-glucuronosyl. 7-MeO-Lut is peak 3. Only the peaks of luteolin, 7-Cl-Lut and 7-MeO-Lut were used when calculating the depletion kinetics.

To follow the metabolization of luteolin, 7-Cl-Lut and 7-MeO-Lut we incubated the compounds with human liver microsomes (Fig 6). The UHPLC chromatograms show that three metabolites are formed when luteolin is treated with the microsomes (Fig 6A). The peaks correspond to the luteolin monoglucuronosyls: luteolin-7-O-glucuronosyl, luteolin-4’-O-glucuronosyl, and luteolin-3’-O-glucuronosyl, and the identification is based on previous LC-MS and NMR speciation [24]. The chromatograms of 7-Cl-Lut and 7-MeO-Lut are shown in Fig 6B and 6C respectively. No analysis of peak 1 and 2 in Fig 6B and 6C was performed, but based on the retention times from a previous investigation [24] peak 1 and 2 in 6B is most likely luteolin-Cl-4’-O-glucuronosyl and luteolin-Cl-3’-O-glucuronosyl respectively. Peak 1 and 2 in 6C is most likely luteolin-MeO-4’-O-glucuronosyl and luteolin-MeO-3’-O-glucuronosyl respectively. Complete determination of the metabolic peaks was not necessary because only the peaks of luteolin, 7-Cl-Lut and 7-MeO-Lut were used when calculating the depletion kinetics. The identification of luteolin, 7-Cl-Lut and 7-MeO-Lut was determined by comparison with the retention time for the untreated drug. The chromatograms in Fig 6B and 6C indicates, as expected, that the chlorine and the methoxy group at the 7-position are inert to glucuronidation.

### Depletion kinetics of luteolin, 7-Cl-Lut and 7-MeO-Lut

**Fig 7:**
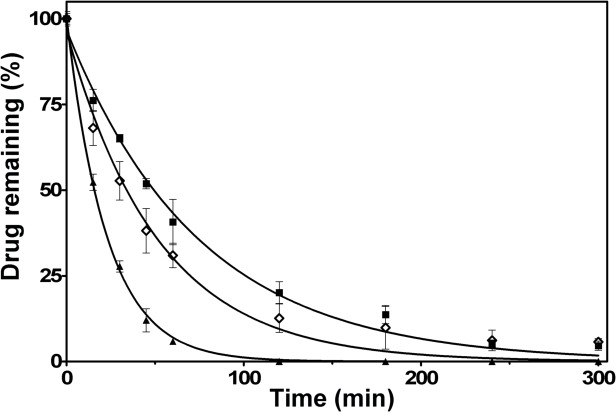
Depletion profile of 10 μM luteolin (triangle), 7-Cl-Lut (square) and 7-MeO-Lut (diamond) after incubation in the presence of 0.1 mg/mL human liver microsomes for various times. Each time point represents the average of three independent samples (mean ± SD) acquired through plotting the integral of the corresponding peaks from the UHPLC analysis.

The metabolization of luteolin, 7-Cl-Lut and 7-MeO-Lut by the microsomes can be followed by the decrease in the substrate concentration over time. The data shown in Fig 7 were fitted to a single-component exponential decay model and the half-lives were determined using Eqs (1) and (2). Luteolin was metabolized about 3-fold faster than 7-Cl-Lut with half-lives of 16 ± 1 min and 52 ± 7 min, respectively. The half-life of 7-MeO-Lut was 36 ± 8 min, about twice as long as luteolins.

### Plasma selectivity is decreased for 7-Cl-Lut and 7-MeO-Lut as compared to luteolin

**Fig 8:**
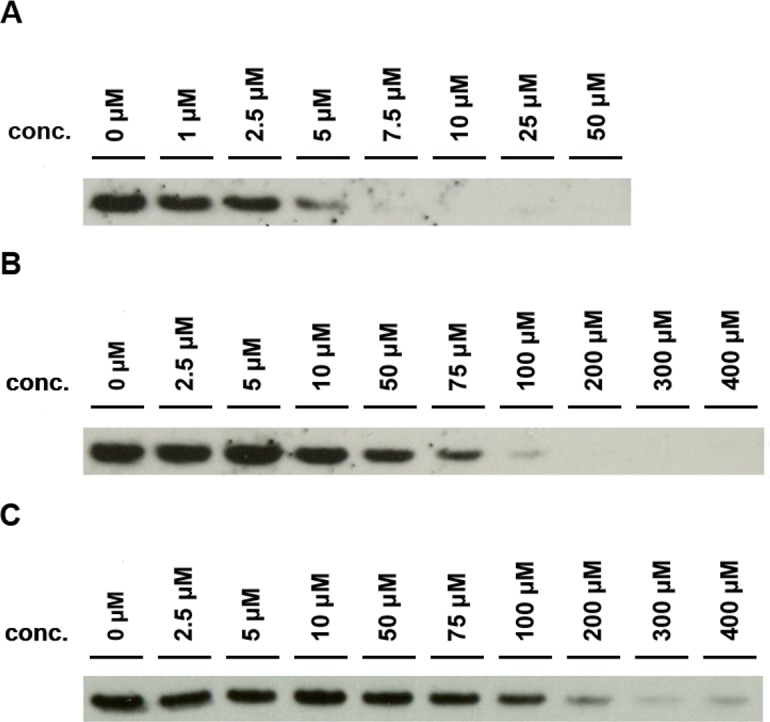
Determination of IC_50_ for luteolin, 7-Cl-Lut and 7-MeO-Lut in human plasma. The drug’s ability to selectively stabilize TTR in plasma was evaluated using a urea denaturation assay [25]. The dissociation of the TTR tetramer is indicated by the presence of a monomeric TTR band on a western blot. (A) Gel-shift assay of various concentrations of luteolin in human plasma. The IC_50_ is ~5 μM. (B) Gel-shift assay of various concentrations of 7-Cl-Lut in human plasma. The IC_50_ is ~65 μM. (C) Gel-shift assay of various concentrations of 7-MeO-Lut in human plasma. The IC_50_ is ~105 μM.

The plasma concentration of TTR is approximately 5 μM [38], and this implies that a drug must be present at ≥5 μM to work effectively as a stabilizer. Here we used a recently developed urea-mediated denaturation assay to determine the drug concentration needed in plasma to obtain the half maximal inhibitory concentration (IC_50_) [29]. In the experiment, the dissociation of TTR into monomers is monitored by the appearance of a monomeric band on a western blot (Fig 8). The fraction of denaturation was quantified by densitometric analysis using ImageJ 1.48 software. Luteolin is a highly selective stabilizer of TTR with an IC_50_ of ~5 μM, 1:1 stoichiometric ratio between drug and tetramer if assuming the plasma concentration to be 5 μM [29]. 7-Cl-Lut, however, lost much of luteolins selectivity and displays an IC_50_ corresponding to around 65 μM (Fig 8B). 7-MeO-Lut also display a significant degree of unspecific binding as compared to luteolin with an IC_50_ corresponding to around 105 μM (Fig 8C).

## Discussion

The discoveries that the dissociation of TTR into monomers is required for amyloid fibril formation [7,8] and that small-molecule ligands are able to increase the activation energy associated with tetramer dissociation [11,12] have paved the way for a novel therapeutic approach. A wide range of TTR ligands have been discovered [11,39–50], and a few have proven beneficial in the treatment of FAP [15,17]. As with all drugs, their effects can be compromised through unspecific binding to other components that both reduce their effective concentrations and trigger unwanted side effects. We have recently discovered that the plant-derived flavonoid luteolin exhibits a very high selectivity for TTR in human plasma [29]. Taken together with its low incidence of side effects, luteolin is an interesting drug candidate for stabilizing the tetrameric structure of TTR. Unfortunately it has rather low metabolic stability *in vivo*, and it is rapidly biotransformed by phase II enzymes with glucuronidation and sulfation as the main modifications. The preferred site for glucuronidation by the liver is the 7-OH position, and glucuronidation at this position renders the molecule unable to stabilize TTR [51]. In order to prevent this, we modified the 7-OH group through replacements with a chloro or a methoxy group that both are inert towards glucuronidation. TTR has two binding pockets, and both T4 and luteolin exhibit negative cooperativity in binding to TTR where the low-affinity site has a binding constant approximately 100 times above the high-affinity interaction. We therefore only focus on the high-affinity site, which in essence dominates the stabilizing effect. The results of the MST binding-assay show that both 7-Cl-Lut and 7-MeO-Lut have lost some of their binding affinity compared to the parental luteolin structure, but they still represent high-affinity binders to TTR. The crystal structure of 7-Cl-Lut complexed to TTR shows only minor differences in protein-ligand interactions compared to the structure with luteolin. Luteolin, with its hydroxyl groups at position 5 and 7, can simultaneously form hydrogen bonds over the tetramer interface (Fig 5C). However, as a consequence of exchanging the 7-OH group for a chloro group, hydrogen bonds to Ser117 and Thr119 side chains on one side of the binding site are lost, which likely explains the observed decrease in binding affinity (compare Fig 5A and 5C). Interestingly, inspection of the hydrogen bond pattern further shows that binding of 7-Cl-Lut at the AA’ and BB’ channel is coupled. That is, if a 7-Cl-Lut ligand binds in the AA’ site, the orientation of the second 7-Cl-Lut ligand at the BB’ site is restricted by the hydrogen-bonding pattern created by the first 7-Cl-Lut ligand (Fig 5A). The 7-MeO-Lut structure showed a reverse binding of the luteolin analogue in the hormone binding pocket (Fig 4D). This is consistent with the orientation observed for several glucuronidated polyphenol metabolites [52]. However, whereas the A ring and the sugar moieties were flexible in the previous structures [52], the A ring with its 7-methoxy group is well defined in the electron density (Fig 4D). The two hydroxy groups at the C ring of 7-MeO-Lut interact with Ser117 and Thr119 but do not provide stability over the tetramer interface (Fig 5B). However, the methoxy group forms hydrophobic contacts with Thr106 and Val121 in the symmetry-related dimer, and in combination with the hydrogen bonds formed, stability over the tetrameric interface is provided also by bound 7-MeO-Lut (Fig 5B).

The persistence of a drug against metabolic activity in the body to large extent determines its efficacy. Luteolin unfortunately has a rather short half-life of around 8 hours [53] mainly due to modification of the 7-OH group of its aromatic A-ring. To study whether the relative persistence in the presence of liver enzymes can be modified as a result of exchanging the 7-OH group for an inert atom, we used an *in vitro* assay based on human hepatic microsomes. Through this approach, the relative resistance of the modified luteolin to glucuronidation by the UGT enzymes could be compared to luteolin [54]. The results showed that chlorination of the 7 position of luteolin increased the metabolic stability of luteolin approximately 3-fold relative to luteolin while addition of a methoxy group doubled the resistance. The activity of the luteolin analogues is still lost over time because the OH groups at the 3' and 4' positions are also subject to conjugation by the UGT enzymes in the liver, they are just not modified as rapidly as the OH group at position 7. Based on these results, the half-life of the 7-Cl-Lut analogue is within an interesting range for a therapeutic ligand. However, the prime reason why luteolin has garnered interest as a TTR-stabilizing agent is its remarkable selectivity in plasma. Addition of an essentially stoichiometric amount of luteolin, relative to the tetrameric concentration of TTR in plasma, results in an almost complete stabilizing effect, which is significantly more efficient compared to several other well-known TTR ligands that sometimes require a more than 40-fold excess to achieve the same stabilizing effect [29]. Using this assay with 7-Cl-Lut showed that the exchange of the 7-OH group for a chlorine affected the selectivity in plasma, and compared to luteolin 7-Cl-Lut requires a 13-fold stoichiometric excess relative to TTR in human plasma to achieve the same stabilizing effect. The 7-MeO-Lut also displays a high degree of unspecific binding to other plasma components and requires 21-fold stoichiometric excess relative to TTR in human plasma to achieve the same stabilizing effect.

The results of the present study show that it is possible to improve the metabolic stability of luteolin by exchanging the OH group at position 7 of the aromatic A-ring with an inert atom. Although the binding strength can be largely preserved, the selectivity in human plasma is diminished to a large extent. We have recently shown how the enthalpic component of binding largely affects selectivity [29], and the finding that removing the ability to form hydrogen bonds between the 7-OH group on luteolin and Ser117 and Thr119 of TTR also lowers the selectivity is therefore in good accordance with the previous study. In this context, it is of interest to discuss the effect of different modifications, i.e. exchange of a hydroxyl group for an inert atom. Considering the 7-OH group, we are limited to a confined space within the structure. Introducing a fluorine would give the opportunity for hydrogen bonding but the C–F group is a very weak hydrogen bond acceptor [55,56]. A structural study on complexes between TTR and ligands having a fluorine within hydrogen-bonding distance of Ser117 and Thr119 shows that the fluorine is not involved in hydrogen bonding but rather is outcompeted in favor of hydrogen bonds between the side chains of Ser117 and Thr119 [57,58].

Based on the structure of TTR, a glucuronid modification of position 3' and 4' in the aromatic B-ring is also not compatible with the interaction between luteolin and TTR. Flavonoids hydroxylated in different positions of the aromatic B-ring have previously been investigated regarding their ability to bind TTR [49], and it can be concluded that this also can have a large influence on the binding strength. According to this previous study, the flavonoid chrysin that lacks the -OH groups in the B-ring and apigenin which only retains a single -OH group in position 4' exhibit 17 and 5 times lower affinity, respectively, compared to luteolin, and this supports the additional importance of the -OH groups in the B-ring of the molecule [49]. Exchanging the hydroxyl groups in the B-ring for a fluoride atom would, however, most likely not result in the generation of new hydrogen bonds due to its poor properties as a hydrogen bond acceptor. Iodine analogues were not pursued at all due to their potential risk to become a thyroxine agonist.

Taken together, the present work pinpoints the flavonoid luteolin as an interesting molecule in the quest to find ligands with the ability to stabilize TTR. We show how luteolin’s resistance against glucuronidation can be modulated and how its half-life *in vivo* might be improved. We specifically show that the luteolin analogues only modestly affects the binding affinity to TTR but has a considerably larger effect with respect to drug selectivity. The ability of Ser117 and Thr119 to accommodate a wide range of ligands by a simple change of their side-chain rotamers, provides one explanation to the maintained high affinity of the luteolin analogues to TTR *in vitro*. We also demonstrate how MST can be used as a simple approach to studying TTR-ligand interactions and how the combined use of liver microsomes and a urea denaturation gel-shift assay provides an efficient method to screen for novel TTR ligands and to test their potential efficacy *in vivo*.
